# The dynamic nexus: exploring the interplay of BMI before, during, and after pregnancy with Metabolic Syndrome (MetS) risk in Chinese lactating women

**DOI:** 10.1186/s12889-023-17344-6

**Published:** 2023-12-05

**Authors:** Liangxia Chen, Jie Ma, Guanmin Su, Longlong Yin, Xiuyu Jiang, Xiangxiang Wang, Lele Liu, Xiaofei Zhang, Xiaohui Xu, Suyun Li, Gaohui Zhang, Ran Zhao, Lianlong Yu

**Affiliations:** 1 Shandong Center for Disease Control and Prevention, Ji’nan, Shandong China; 2 Health Management Center, Central Hospital Affiliated to Shandong First Medical University, Ji’nan, Shandong China; 3 Department of Gynecology, Central Hospital Affiliated to Shandong First Medical University, Ji’nan, Shandong China

**Keywords:** Metabolic syndrome, Lactation women, Body mass index (BMI), Pre-pregnancy, Pregnancy

## Abstract

**Background and aim:**

The health implications of BMI and MetS in lactating women are significant. This study aims to investigate the relationship between risk of Mets in lactation and BMI in four stages: pre-pregnancy, prenatal period, 42 days postpartum, and current lactation.

**Methods and results:**

A total of 1870 Lactating Women within 2 years after delivery were included from "China Child and Lactating Mother Nutrition Health Surveillance (2016–2017)". Logistic regression model and Restricted cubic spline (RCS) were used to estimate the relationship between BMI and risk of MetS. ROC analysis was used to determine the threshold for the risk of MetS. Chain mediating effect analysis was used to verify the mediating effect. BMI of MetS group in all stages were higher than non-MetS group (*P* < 0.0001). There were significant positive correlations between BMI in each stage and ORs of MetS during lactation (*P* < 0.05). The best cut-off values for BMI in the four stages were 23.47, 30.49, 26.04 and 25.47 kg/m^2^. The non-linear spline test at BMI in 42 days postpartum, current and MetS in lactation was statistically significant (*P* non-linear = 0.0223, 0.0003). The mediation effect of all chains have to work through lactation BMI. The total indirect effect accounted for 80.95% of the total effect.

**Conclusions:**

The risk of MetS in lactating women is due to a high BMI base before pregnancy and postpartum. High BMI in all stages of pregnancy and postpartum were risk factors for MetS in lactation. BMI during lactation plays a key role in the risk of MetS.

## Introduction

The well-being of women during pregnancy, the postpartum period, and lactation is a matter of global concern [1]. To attain an optimal nutritional status, lactating women should enhance their dietary intake [2]. Paradoxically, an excessive energy intake may lead to the accumulation of body fat and an increased risk of developing overweight or obesity, presenting a challenge in regaining pre-pregnancy weight [3]. Overweight and obesity are significant worldwide public health issues and are not only strongly linked to pregnancy [4] but also to various chronic diseases. It is now firmly established that being overweight or obese increases the susceptibility to metabolic conditions such as dyslipidemia, hypertension, and type 2 diabetes, and is also closely associated with metabolic syndrome (MetS) [5, 6].

MetS is a complex group of metabolic disorders including abdominal obesity, hypertension, hypertriglyceridemia, low HDL cholesterol, and elevated fasting glucose. The prevalence of MetS is steadily increasing worldwide [7]. Also MetS is very common in overweight and obese individuals [8]. Maternal obesity can be considered as a risk factor for poor breastfeeding outcomes [9]. Pre-pregnancy body mass index (BMI) and weight gain during pregnancy are strongly associated [10]. BMI is the simplest, practical and commonly used tool to assess overweight and obesity. Studies have shown a strong relationship between waist circumference (WC) and BMI in women, and BMI is more clinically available and commonly used in the assessment of MetS compared to WC [11, 12]. According to a previous study, MetS was more common in women than in men [13]. The impact of childbirth on maternal metabolism is significant. For example, the resulting increased risk of central obesity and the higher the number of births causes a higher risk of MetS [14]. Lactation is a transitional phase for women to return from pregnancy to normal physiological status. However, not only is there a paucity of research on MetS during pregnancy [15], but studies on the relationship between BMI at various stages of pregnancy and MetS in lactating women have not been reported. BMI has the potential to induce disturbances in inflammation and the immune system, both of which are pivotal factors contributing to the pathogenesis of Metabolic Syndrome (MetS) [14].

In previous research, it has been unequivocally established that overweight and obesity play pivotal roles as risk factors for the development of metabolic syndrome (MetS). However, it is worth noting that there is currently a conspicuous dearth of studies that specifically address the validation and in-depth exploration of this relationship in lactating women, a population characterized by unique physiological conditions. A significant knowledge gap persists in this area, necessitating further investigations to elucidate the association between overweight and obesity and MetS in lactating women. Such endeavors hold promise for the development of targeted strategies and interventions to enhance the health management of this specific population, contributing to the advancement of women's health during the lactation period. Therefore, this study intends to investigate the relationship between BMI changes and the risk of developing Mets during lactation, starting from the four stages of BMI: pre-pregnancy, prenatal period, 42 days postpartum, and lactation, to explore new ideas for the prevention and control of MetS in women of childbearing age.

## Methods

### Study population

A total of 1870 lactating women within 2 years after delivery were included in this study. Data for this study were obtained from the "China Child and Lactating Mother Nutrition Health Surveillance (2016–2017)" approved by the Chinese Center for Disease Control and Prevention (CDC). Three provinces, Shandong, Hebei and Guizhou, located in East China, Central China and West China, were selected for the sample survey.The data is from monitoring database. The purpose of the survey was to understand the health status of lactating women, to take into account the comprehensive health problems of the respondents. In the absence of authoritative data regarding the prevalence of Metabolic Syndrome (MetS), the calculation of sample size relied upon the anemia rate as a reference standard. Chinese lactating mother anemia rate of 9.3% in 2013 was taken as the calculated marker to determine the sample size [16]. The relative standard error was controlled to be within 15% by taking r = 15% and $\updelta$ = 15% × 9.3% to ensure a precision of 1.395%. The confidence level was taken as 95% (two-sided), i.e. μ = 1.96. Sample size calculation formula,

$$\mathrm{n}=\frac{{\upmu }_{\mathrm{\alpha }/2}^{2}*\mathrm{p}\left(1-\mathrm{p}\right)}{{\updelta }^{2}}$$

**Fig. 1:**
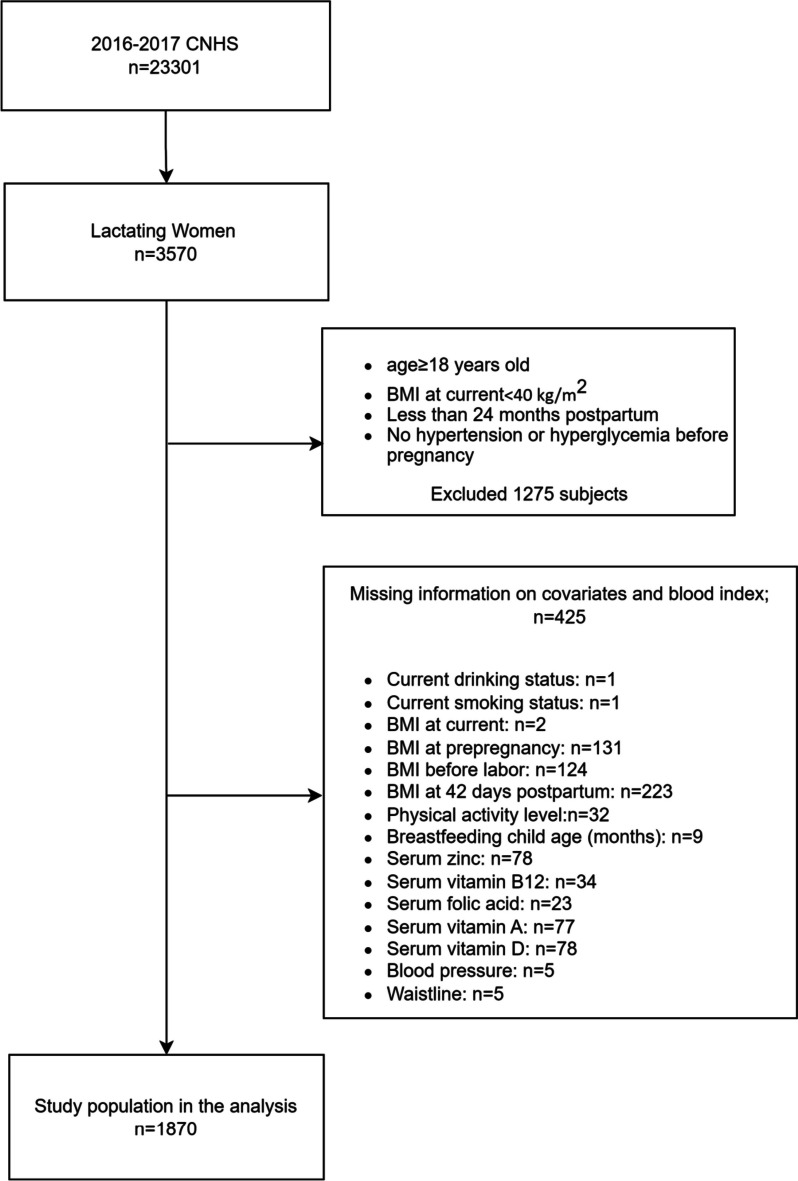
Flowchart of study selection

Accordingly, the sample size of the survey should not be less than 1665 people. Thirty-five districts or counties were randomly selected based on geographic location. Two townships (streets) were selected from each district or county, two village (neighborhood) committees were selected from each township (street), and at least 10 lactating women aged 18–50 years with breastfeeding children under 2 years old were selected from each village (neighborhood) committee. Subjects with major diseases such as hypertension, diabetes and oncology were excluded from the study. The survey process used a tablet with a quality control program to administer the questionnaire. Figure 1 displays the flow chart illustrating the process of subject inclusion and exclusion.

#### Inclusion criteria

1. Lactating Women Aged ≥ 18 Years: we included lactating women who were 18 years of age or older to ensure that the subjects were adults.
2. Duration of Lactation < 24 Months: Lactating women with a lactation duration less than 24 months were included to focus on the early stages of lactation.
3. BMI < 40 kg/m^2^: we considered lactating women with a body mass index (BMI) below 40 kg/m^2^ to maintain a range of BMI values within reasonable limits for the study.
4. Having Complete Information on MetS Components, BMI, and Covariates: Subjects needed to have comprehensive data on Metabolic Syndrome (MetS) components, BMI, and other relevant covariates for the study. This ensured the availability of essential data for a thorough analysis.

#### Exclusion criteria

1. Clinically Significant Disease or Disability: We excluded individuals with clinically significant diseases or disabilities to maintain a sample of participants without confounding health issues that could influence the study's outcomes.
2. Individuals with pre-pregnancy hyperglycemia and hypertension were also excluded based on questionnaires.

This study was conducted according to the guidelines laid down in the Declaration of Helsinki and all procedures involving human subjects were approved by the the Ethics Committee of the National Institute for Nutrition and Health, Chinese Center for Disease Control and Prevention, and the ethical approval number obtained was 201,614. Written informed consent was obtained from all subjects.

### Data collection

The data sources collected for this survey included questionnaires, physical examinations and laboratory tests. In order to ensure the precision and uniformity of our data, we employed a questionnaire meticulously curated by experts from the Chinese Center for Disease Control and Prevention. This questionnaire was administered through one-on-one, face-to-face interviews conducted by professional surveyors. This comprehensive survey encompassed the collection of demographic data, physical activity patterns, dietary habits, smoking and alcohol status, as well as an in-depth exploration of the participants' medical histories. This methodological approach guarantees the acquisition of exhaustive and detailed information, facilitating a more profound comprehension of the participants' backgrounds and potential influencing variables. The national and provincial working groups were responsible for quality control of the survey. District and county CDC staff trained by the local CDC were responsible for fasting blood sample collection, interviews and questionnaires. To ensure the quality of the data, subjects were informed in advance to perform the physical examination in the early morning of the second day after an overnight fast while avoiding heavy clothing and shoes. The current BMI of lactating women was calculated after the survey with the formula weight (kg)/square of height (m^2^). Also, physical measurement instruments were purchased and calibrated uniformly at all survey sites: an electronic weight scale (G&G TC-200 K) and an electronic sphygmomanometer (OMRON HBP1300) with a measurement accuracy of 0.1 cm, 0.1 kg and 1 mmHg, respectively.

The pre-pregnancy weight was obtained from the medical examination card within six months prior to pregnancy or within 1–2 months of pregnancy, or from your own recollection to the nearest 0.1 kg. The prenatal weight was obtained from the health care card or from recollection of the prenatal weight to the nearest 0.1 kg. The post-pregnancy weight at 42 days was obtained from the health care card or from recollection of the post-pregnancy weight at 42 days to the nearest 0.1 kg.

Staff collected fasting venous blood samples from subjects early in the morning, separated the plasma within 1 h, and sent it to the laboratory via cold chain, refrigerated at -80 °C. Fasting blood glucose, hemoglobin, serum vitamin B12, zinc, vitamin A, vitamin D, hs-CRP, transferrin receptor, ferritin, albumin, and total protein were measured using a Hitachi Autoanalyzer 7600 (Hitachi, Tokyo, Japan). All measurements are performed by professional laboratory personnel and under strict quality control in the laboratory.

The definition of MetS is based on the International Diabetes Federation (IDF) Task Force on Epidemiology and Prevention Guidelines [17]: Abdominal obesity: Chinese women are defined by the cut-off point by WC ≥ 80 cm or BMI > 30 kg/m^2^; and the presence of two or more of the following clinical features: (1) TG ≥ 1.7 mmol/L; (2) HDL-C < 1.29 mmol/L; (3) Systolic blood pressure ≥ 130 mmHg and/or diastolic blood pressure ≥ 85 mmHg; (4) FPG ≥ 5.6 mmol/L.

### Covariates

The covariates involved in the analysis of this study included age, parity, breastfeeding child age (months), current smoking status, current drinking status, physical activity level, FPG, history of gestational diabetes, history of gestational hypertension and hemoglobin. These covariates were collected using a standardized questionnaire and physical examination of a uniform design.

Also, to address possible bias in the analysis, models were used in the study to correct for the corresponding covariates. Models were adjusted for age, age of children (months), physical activity level and current smoking status. current alcohol drinking status, parity, history of gestational diabetes, history of gestational hypertension, and hemoglobin.

### Statistical analysis

The chi-square test (categorical variable) and t-test (continuous variable) were used to assess the differences between MetS and non-MetS groups in basic characteristics and blood biochemical indices. Heat maps and hierarchical clustering were used to describe the relationship between BMI and blood biochemical indicators at different time periods. Multiple logistic regression models were used to estimate the relationship between BMI and the risk of developing MetS and its components at different periods. Also logistic regression models were adjusted for potential confounders including age, age of children (month), physical activity level, current smoking status, current alcohol drinking status, parity, history of gestational diabetes, history of gestational hypertension, and hemoglobin. According to the central limit theorem, the distribution of each variable tended to be normally distributed in this study because of its large sample data. For sensitivity analysis, we stratified the data by age, age of children (months), parity, physical activity level, and hemoglobin. Multiplicative interaction analyses were also performed to assess the effect of stratification factors on the relationship between BMI and the risk of MetS at each stage. In addition, restricted cubic spline (RCS) was used to explore the nonlinear relationship between BMI and ORs of MetS at each stage, and three nodes were selected for curve fitting based on the AIC optimality principle. Meanwhile, ROC analysis was used to determine the minimum threshold for the risk of MetS prevalence due to each stage of BMI, and the cutoff value for differentiating MetS by each stage of BMI was selected using the Youden index criterion [18]. In addition, chain mediating effect analysis was used to verify the mediating effect of pre-pregnancy, prenatal, 42 days postpartum and lactation periods contributing to the risk of MetS due to BMI. Statistical analysis was performed using R 4.1.2. *P* < 0.05 (two-tailed) was considered significant.

## Results

**Fig. 2:**
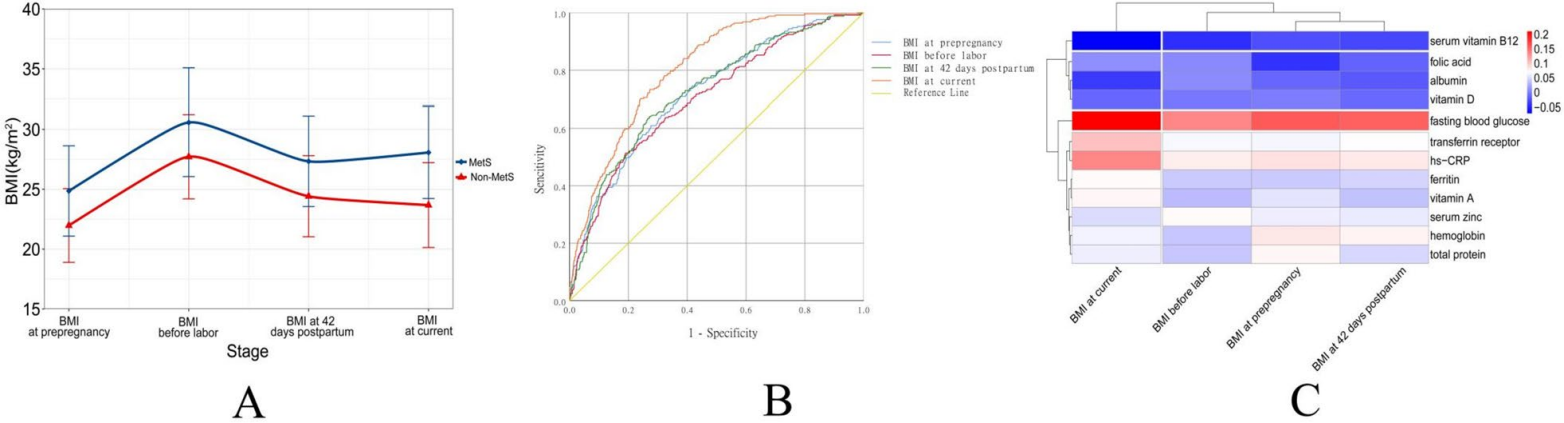
**A** Change in BMI with different stage for MetS and Non-MetS groups. **B** Receiver operator characteristic (ROC) curves for BMI at prepregnancy, BMI before labor, BMI at 42 days postpartum, BMI at current to predict Metabolic Syndrome. Area under ROC curve = 0.724, 0.706, 0.728, 0.806 (*P* < 0.0001). The best cut-off value for BMI at prepregnancy, BMI before labor, BMI at 42 days postpartum, BMI at current were 23.47, 30.49, 26.04, 25.47 kg/m^2^ according to the Youden Index standard. **C** Clustering and correlation heat map between BMI stage and blood biochemical indexes

The mean age of the 1870 lactating women was 29.86 years with a pre-pregnancy BMI of 22.36 ± 3.33 kg/m^2^, a pre-partum BMI of 28.10 ± 3.79 kg/m^2^, 42 days postpartum BMI of 24.81 ± 3.58 kg/m^2^ and a current lactation BMI of 24.27 ± 3.89 kg/m^2^. The changes in BMI of the MetS and non-MetS groups during pregnancy and lactation are shown in Fig. 2(A), and there was a statistical difference in BMI at all stages between the two groups (*P* < 0.0001) (Table 1). The mean age of lactating children was 7.92 ± 5.01 months. There were 660 (35.29%) participants with 1 parity and 1124 (60.11%) with 2 parities. Light manual workers accounted for 1822 (81.39%), medium manual laborers 340 (18.18%). There were 251 subjects with MetS, while 983 subjects with abdominal obesity, 292 with high BP, 243 with high TG, 555 with low HDL, and 188 with elevated FG.

**Table 1:** Demographic and clinical characteristics of MetS and Non-MetS groups

	**MetS** **(*n* = 251)**	**Non-MetS** **(*n* = 1619)**	t/χ^2^	*P-value*
Age (years)	31.75(5.72)	29.57(5.14)	5.70	< 0.0001
BMI at prepregnancy	24.86(3.77)	21.97(3.08)	11.54	< 0.0001
BMI before labor	30.58(4.51)	27.71(3.51)	9.61	< 0.0001
BMI at 42 days postpartum	27.33(3.78)	24.42(3.39)	11.54	< 0.0001
BMI at current	28.07(3.85)	23.68(3.54)	18.07	< 0.0001
Parity numbers	1.81(0.65)	1.68(0.56)	2.90	0.0040
Age of children (mouths)	8.36(5.24)	7.85(4.99)	1.50	0.1332
Drinker (%)	5 (1.99)	33 (2.04)	0.0023	0.9614
Smoker (%)	2(0.80)	10(0.62)	0.1094	0.7408
Physical activity level (%)				
Light	206(10.97)	1316(70.07)	1.2671	0.5307
Moderate	45(2.40)	295(15.71)		
Vigorous	0	8(0.43)		
History of gestational diabetes (%)	19(7.57)	63(3.89)	7.0130	0.0081
History of gestational hypertension (%)	18(7.17)	37(2.29)	18.1728	< 0.0001
TC (mmol/L)	4.65(0.97)	4.28(0.84)	5.63	< 0.0001
TG (mmol/L)	2.07(1.14)	0.91(0.48)	15.80	< 0.0001
HDL (mmol/L)	1.15(0.23)	1.56(0.34)	-24.25	< 0.0001
LDL (mmol/L)	2.76(0.89)	2.45(0.75)	5.19	< 0.0001
Serum zinc (μg/dL)	3.93(14.55)	2.91(12.12)	1.05	0.2939
Blood glucose (mmol/L)	5.38(1.29)	4.81(0.59)	6.85	< 0.0001
Ferritin (ng/mL)	63.75(49.55)	48.42(38.99)	4.68	< 0.0001
Transferrin receptor (mg/L)	3.43(1.34)	3.3(1.5)	1.42	0.1551
Hemoglobin (g/L)	135.39(11.83)	131.47(13.39)	4.80	< 0.0001
Serum Vitamin B12 (pg/mL)	454.72(200.44)	462.3(235.21)	-0.54	0.5867
Serum folic acid (ng/mL)	7.75(23.76)	24.92(106.02)	-5.66	< 0.0001
Hs-CRP (mg/L)	2.49(3.03)	1.65(4.36)	3.83	< 0.0001
Albumin (g/L)	46.97(3.16)	47.64(3.41)	-2.92	0.0036
Total protein (g/L)	75.64(5.26)	76.06(5.14)	-1.22	0.2243
Serum Vitamin A (µmol/L))	0.52(0.16)	0.48(0.27)	3.80	0.0002
Serum Vitamin D (ng/mL)	17.28(5.52)	17.25(6.28)	0.08	0.9336
Systolic pressure (mmHg)	127.07(14.14)	115.51(10.72)	12.41	< 0.0001
Diastolic pressure (mmHg)	78.79(11)	71.05(8.72)	10.64	< 0.0001

*Note*: Data were presented as mean (SD) or n (%)

Table 1 listed the demographic and clinical characteristics of the MetS and non-MetS groups. Compared to the non-MetS group, the MetS group had a higher age, BMI by stage, parity, history of gestational diabetes (%), history of gestational hypertension (%), TC, TG, LDL, blood glucose, serum ferritin, hemoglobin, Hs-CRP, vitamin A, systolic pressure and diastolic pressure (*P* < 0.05). Also, HDL, serum folic acid and albumin were lower in the MetS group than in the non-MetS group (*P* < 0.05).

Table 2 presented the logistic regression results for the association between BMI at different stages and the risk of current MetS lactation. The model found a significant positive association between increasing BMI at each stage and the ORs of both MetS and its components after several adjustments for confounders (*P* < 0.05). The same positive correlation was also found for the difference value between BMI at current and BMI at 42 days postpartum as independent variables (*P* < 0.05). The sensitivity analysis of BMI at each stage on the current risk of MetS prevalence in lactating women was concentrated in Table 3. The results showed that the positive association between BMI at all stages and MetS risk in lactation was almost consistent in all stratified analyses. And there was an interaction of maternal age on the relationship between BMI at 42 days postpartum and MetS risk in lactating women (*P* = 0.0230).

**Table 2:** Association of BMI stage and the prevalence of MetS and its components

	**Abdominal obesity**	**High BP**	**High TG**	**Low HDL**	**Elevated FG**	**MetS**
BMI at prepregnancy						
**Crude OR (95% CI)**	1.459 (1.399, 1.520)	1.171 (1.130, 1.214)	1.149 (1.106, 1.193)	1.133 (1.099, 1.167)	1.141 (1.094, 1.189)	1.270 (1.221, 1.321)
**Adjusted OR**^**a**^** (95% CI)**	1.449 (1.389,1.511)	1.161 (1.119, 1.205)	1.133 (1.090, 1.178)	1.139 (1.104, 1.175)	1.125 (1.078, 1.175)	1.256 (1.206, 1.308)
**Adjusted OR**^**b**^** (95% CI)**	1.452 (1.391, 1.515)	1.141 (1.098, 1.186)	1.132 (1.087, 1.178)	1.138 (1.102, 1.174)	1.109 (1.061, 1.160)	1.243 (1.192, 1.296)
BMI before labor						
**Crude OR (95% CI)**	1.324 (1.280, 1.369)	1.118 (1.081, 1.155)	1.108 (1.071, 1.148)	1.101 (1.071, 1.131)	1.090 (1.050, 1.131)	1.215 (1.171, 1.262)
**Adjusted OR**^**a**^** (95% CI)**	1.318 (1.274, 1.364)	1.111 (1.074, 1.149)	1.099 (1.061, 1.139)	1.102 (1.072, 1.133)	1.082 (1.042, 1.124)	1.208 (1.162, 1.255)
**Adjusted OR**^**b**^** (95% CI)**	1.321 (1.276, 1.368)	1.092 (1.055, 1.131)	1.098 (1.059, 1.139)	1.101 (1.071, 1.133)	1.071 (1.030, 1.114)	1.201 (1.155, 1.249)
BMI at 42 days postpartum						
**Crude OR (95% CI)**	1.435 (1.380, 1.492)	1.129 (1.091, 1.168)	1.133 (1.092, 1.174)	1.111 (1.080, 1.144)	1.110 (1.068, 1.155)	1.245 (1.197, 1.294)
**Adjusted OR**^**a**^** (95% CI)**	1.428 (1.373, 1.486)	1.119 (1.080, 1.158)	1.120 (1.079, 1.162)	1.115 (1.083, 1.147)	1.101 (1.057, 1.146)	1.233 (1.185, 1.283)
**Adjusted OR**^**b**^** (95% CI)**	1.432 (1.375, 1.491)	1.100 (1.061, 1.140)	1.117 (1.076, 1.160)	1.112 (1.080, 1.146)	1.088 (1.043, 1.134)	1.222 (1.173, 1.272)
BMI at current						
**Crude OR (95% CI)**	1.818 (1.720, 1.921)	1.178 (1.141, 1.216)	1.179 (1.140, 1.219)	1.162 (1.131, 1.193)	1.150 (1.109, 1.192)	1.331 (1.281, 1.383)
**Adjusted OR**^**a**^** (95% CI)**	1.810 (1.713, 1.913)	1.173 (1.136, 1.211)	1.172 (1.133, 1.213)	1.167 (1.135, 1.199)	1.143 (1.101, 1.186)	1.330 (1.279, 1.383)
**Adjusted OR**^**b**^** (95% CI)**	1.813 (1.715, 1.916)	1.165 (1.127, 1.204)	1.172 (1.132, 1.213)	1.166 (1.134, 1.198)	1.133 (1.091, 1.176)	1.323 (1.271, 1.376)
△BMI before labor—BMI at prepregnancy						
**Crude OR (95% CI)**	0.993 (0.960, 1.028)	0.969 (0.921, 1.021)	0.990 (0.939, 1.044)	0.993 (0.956, 1.032)	0.966 (0.908, 1.029)	0.997 (0.947, 1.049)
**Adjusted OR**^**a**^** (95% CI)**	1.002 (0.967, 1.038)	0.979 (0.931, 1.030)	1.001 (0.951, 1.053)	0.994 (0.956, 1.032)	0.978 (0.919, 1.040)	1.010 (0.962, 1.060)
**Adjusted OR**^**b**^** (95% CI)**	1.006 (0.971, 1.043)	0.972 (0.921, 1.027)	1.002 (0.952, 1.054)	0.995 (0.958, 1.034)	0.984 (0.927, 1.044)	1.012 (0.965, 1.063)
△BMI at 42 days postpartum—BMI before labor						
**Crude OR (95% CI)**	1.097 (1.042, 1.154)	0.988 (0.923, 1.058)	1.035 (0.961, 1.116)	1.000 (0.947, 1.056)	1.025 (0.943, 1.114)	1.017 (0.945, 1.094)
**Adjusted OR**^**a**^** (95% CI)**	1.083 (1.029, 1.141)	0.974 (0.910, 1.043)	1.022 (0.947, 1.102)	0.999 (0.946, 1.055)	1.013 (0.930, 1.103)	1.000 (0.928, 1.077)
**Adjusted OR**^**b**^** (95% CI)**	1.082 (1.028, 1.140)	0.980 (0.912, 1.052)	1.017 (0.942, 1.098)	0.996 (0.943, 1.052)	1.010 (0.928, 1.099)	0.995 (0.923, 1.072)
△BMI at current -BMI at 42 days postpartum						
**Crude OR (95% CI)**	1.273 (1.219, 1.330)	1.133 (1.083, 1.185)	1.129 (1.076, 1.184)	1.127 (1.084, 1.171)	1.110 (1.053, 1.169)	1.208 (1.150, 1.268)
**Adjusted OR**^**a**^** (95% CI)**	1.276 (1.220, 1.334)	1.140 (1.088, 1.193)	1.136 (1.082, 1.193)	1.129 (1.086, 1.174)	1.117 (1.060, 1.178)	1.222 (1.163, 1.284)
**Adjusted OR**^**b**^** (95% CI)**	1.279 (1.223,1.337)	1.160 (1.106, 1.216)	1.139 (1.084, 1.197)	1.131 (1.088, 1.176)	1.122 (1.064, 1.184)	1.236 (1.174, 1.300)

^a^Model 1, adjusted for age, age of children (month), physical activity level, current smoking status, current alcohol drinking status. ^b^Model 2, adjusted for Model 1, parity numbers, history of gestational diabetes, history of gestational hypertension, hemoglobin

**Table 3:** Stratified analyses of MetS risk and BMI stage by age, age of children (mouths), parity, physical activity level, hemoglobin

	BMI at prepregnancy		BMI before labor		BMI at 42 days postpartum		BMI at current		△BMI before labor—BMI at prepregnancy		△BMI at 42 days postpartum—BMI before labor		△BMI at current -BMI at 42 days postpartum	
	**MetS (OR, 95%CI)**	*P* Value for Interaction	**MetS (OR, 95%CI)**	*P* Value for Interaction	**MetS (OR, 95%CI)**	*P* Value for Interaction	**MetS (OR, 95%CI)**	*P* Value for Interaction	**MetS (OR, 95%CI)**	*P* Value for Interaction	**MetS (OR, 95%CI)**	*P* Value for Interaction	**MetS (OR, 95%CI)**	*P* Value for Interaction
Age ( years)		0.1585		0.1872		0.0230		0.1891		0.3572		0.2554		0.2479
< 30	1.230 (1.155, 1.310)		1.177 (1.111, 1.247)		1.196 (1.127, 1.269)		1.306 (1.233, 1.383)		0.986 (0.900, 1.079)		1.031 (0.914, 1.162)		1.258 (1.171, 1.350)	
≥ 30	1.254 (1.185, 1.327)		1.217 (1.152, 1.286)		1.251 (1.181,1.326)		1.347 (1.272, 1.426)		1.025 (0.967, 1.085)		0.981 (0.889, 1.083)		1.207 (1.121, 1.124)	
Age of children (mouths)		0.9235		0.5235		0.7624		0.5239		0.0438		0.0408		0.0358
< 12 months	1.240 (1.183, 1.301)		1.218 (1.164, 1.275)		1.230 (1.173, 1.289)		1.320 (1.260, 1.382)		1.046 (0.993, 1.101)		0.957 (0.879, 1.041)		1.188 (1.119, 1.262)	
12–23 months	1.257 (1.150, 1.373)		1.159 (1.066, 1.260)		1.217 (1.115, 1.328)		1.365 (1.253, 1.487)		0.883 (0.787, 0.991)		1.128 (0.968, 1.313)		1.364 (1.229, 1.514)	
Parity		0.4000		0.1903		0.1234		0.1383		0.0835		0.9756		0.149
Primiparous	1.254 (1.165, 1.350)		1.241 (1.158, 1.331)		1.257 (1.171, 1.348)		1.363 (1.269, 1.465)		1.083 (0.991, 1.183)		0.966 (0.853, 1.095)		1.183 (1.092, 1.282)	
Multiparous	1.247 (1.185, 1.313)		1.186 (1.130, 1.244)		1.213 (1.153, 1.276)		1.312 (1.249, 1.378)		0.947 (0.880, 1.018)		1.028 (0.936, 1.130)		1.269 (1.187, 1.357)	
Physical activity level		0.3631		0.5838		0.6447		0.8915		0.8935		0.7011		0.6538
Light	1.252 (1.195, 1.311)		1.207 (1.155, 1.260)		1.226 (1.172, 1.282)		1.327 (1.270, 1.387)		1.014 (0.963, 1.067)		0.986 (0.908, 1.070)		1.232 (1.163, 1.305)	
Moderate & Vigorous	1.207 (1.089, 1.338)		1.167 (1.060, 1.284)		1.200 (1.085, 1.328)		1.310 (1.193, 1.439)		0.991 (0.852, 1.153)		1.038 (0.864, 1.248)		1.250 (1.118, 1.397)	
Hemoglobin (g/L)		0.3270		0.2869		0.5624		0.7220		0.1134		0.9527		0.1441
< 133(median)	1.290 (1.206, 1.381)		1.184 (1.116, 1.257)		1.207 (1.134, 1.284)		1.334 (1.255, 1.418)		0.921 (0.834, 1.018)		1.014 (0.894, 1.150)		1.306 (1.207, 1.414)	
≥ 133(median)	1.223 (1.159, 1.290)		1.230 (1.164, 1.300)		1.243 (1.175, 1.314)		1.329 (1.259, 1.403)		1.042 (0.985, 1.102)		0.981 (0.893, 1.077)		1.185 (1.108, 1.267)	

Logistic regression model with adjustment for age, age of children (month), physical activity level, current smoking status, current alcohol drinking status, parity numbers, history of gestational diabetes, history of gestational hypertension, and hemoglobin

**Fig. 3:**
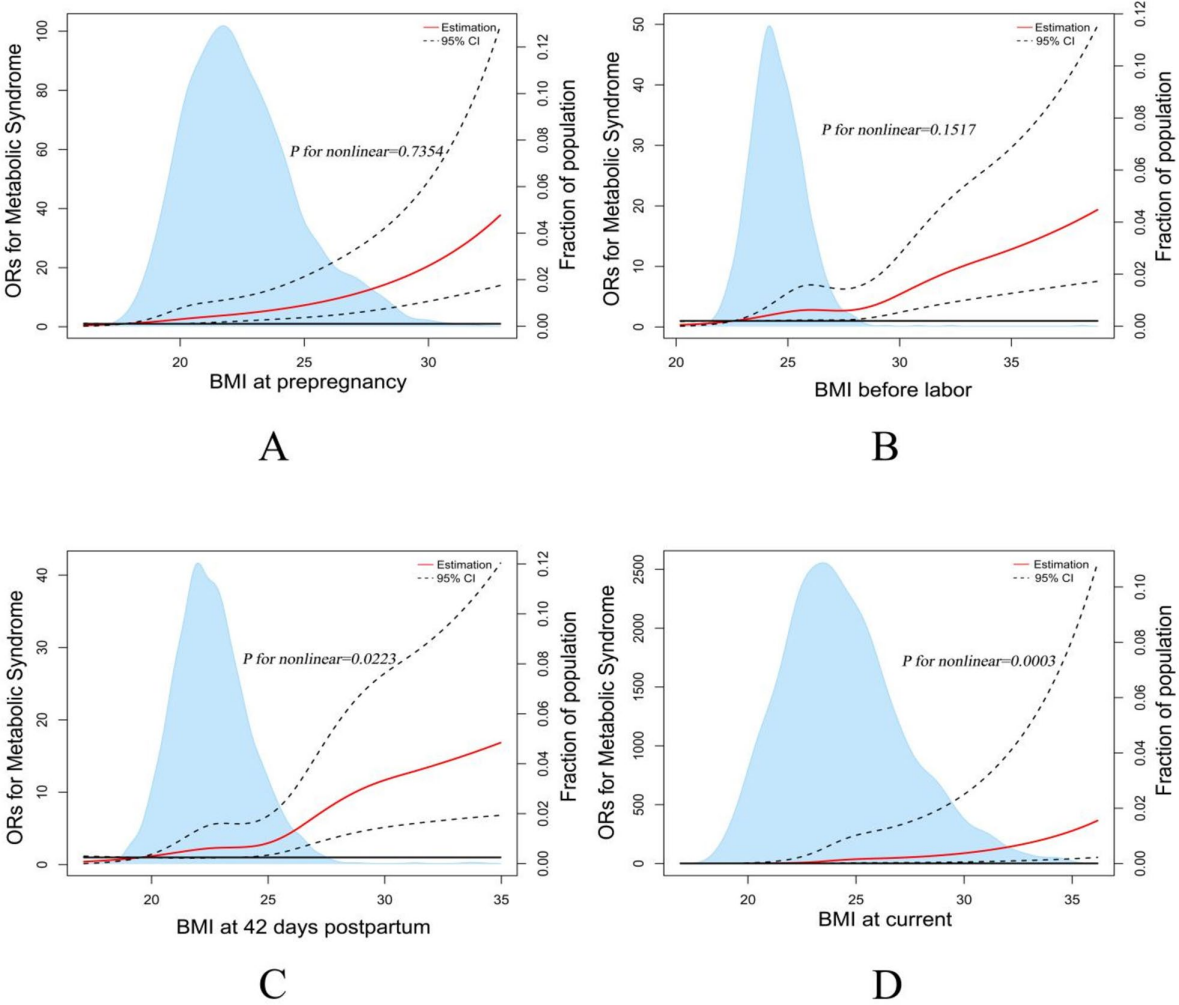
Representation of restricted cubic spline (RCS) logistic regression models. Red Solid line, OR as a function of BMI adjusted for age, age of children (month), physical activity level, current smoking status, current alcohol drinking status, parity numbers, history of gestational diabetes, history of gestational hypertension, and hemoglobin; dashed lines, 95% CIs. **A** RCS logistic regression models for BMI at prepregnancy and OR of Metabolic Syndrome (*P* for nonlinear trend = 0.7354). **B** RCS logistic regression models for BMI before labor and OR of Metabolic Syndrome (*P* for nonlinear trend = 0.1517). **C** RCS logistic regression models for BMI at 42 days postpartum and OR of Metabolic Syndrome (*P* for nonlinear trend = 0.0223). **D** RCS logistic regression models for BMI at current and OR of Metabolic Syndrome (*P* for nonlinear trend = 0.0003)

Using ROC curve analysis, the cut-off values for differentiating MetS at each stage of pregnancy and lactation were obtained based on sensitivity, specificity and Youden Index standard (Fig. 2B). The best cut-off values for BMI at pre-pregnancy, BMI before labor, BMI at 42 days postpartum, BMI in lactation were 23.47, 30.49, 26.04 and 25.47 kg/m^2^, according to the Youden Index standard. The heat map and hierarchical clustering of BMI and blood indicators for women at each stage of pregnancy and lactation are shown in Fig. 2C. Serum vitamin B12, folic acid, albumin, and vitamin D were grouped together with each stage of BMI. Transferrin receptor, hs-CRP, ferritin, vitamin A, serum zinc, hemoglobin, and total protein were grouped together. Transferrin receptor, hs-CRP, ferritin, vitamin A, serum zinc, hemoglobin, and total protein were grouped into one category. Fasting blood glucose was grouped into one category. The different stages of BMI, BMI at pre-pregnancy, BMI before labor, and BMI at 42 days postpartum were grouped into one category, and BMI at current was a separate category. In the logistic regression model-based RCS, ORs for MetS increased significantly with increasing BMI in women of reproductive age at all stages (Fig. 3). The non-linear spline test for BMI at 42 days postpartum, current BMI and MetS during lactation was statistically significant (*P* non-linear = 0.0223, 0.0003).

**Fig. 4:**
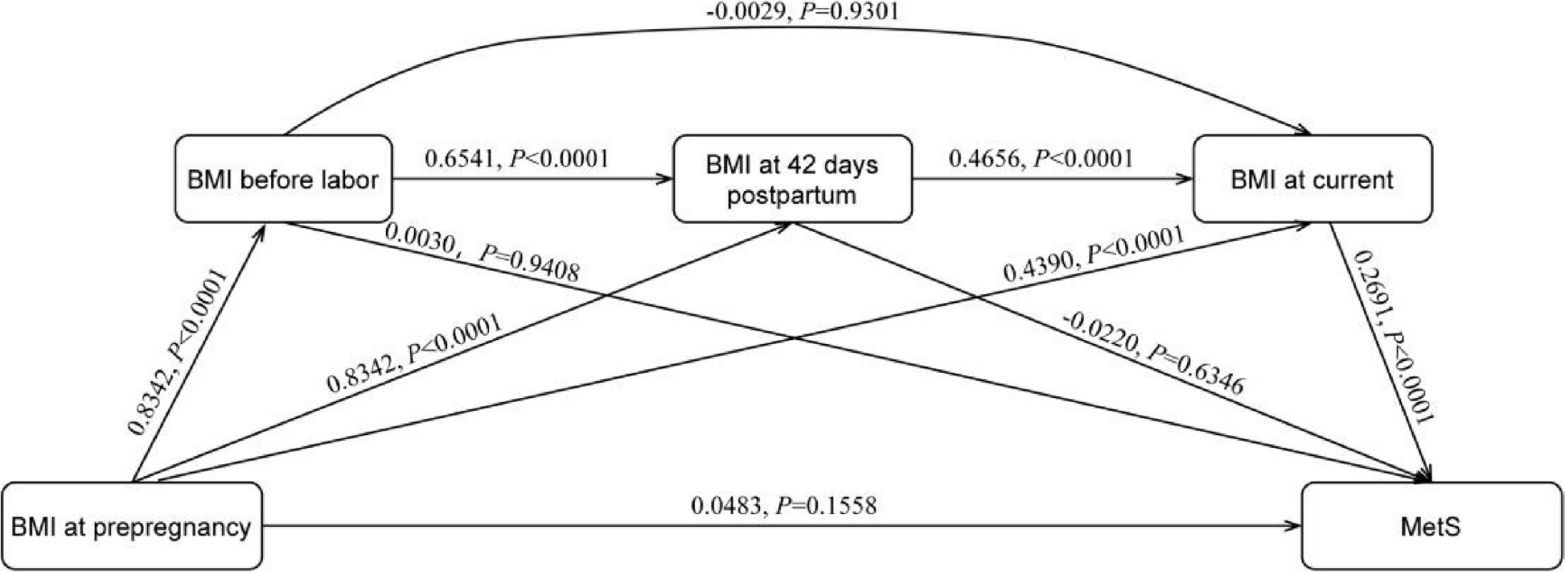
Hypothesis and results of chain mediation effect of pre-pregnancy, pregnancy and lactation BMI on MetS

Figure 4 demonstrates the chain mediation effect and path coefficients of BMI at pre-pregnancy on the relationship between BMI at pre-pregnancy and MetS during pregnancy and lactation. The mediation effect arises through three chains of mediation: first, the indirect effect consisting of BMI at pre-pregnancy → BMI before labor → BMI at 42 days postpartum → BMI at current → MetS with Bootstrap 95% confidence interval [0.0501,0.0931], indicating that the chain mediating effect of BMI before labor, BMI at 42 days postpartum, and BMI at current was significant; second, the indirect effect consisting of BMI at pre-pregnancy → BMI at 42 days postpartum → BMI at current → MetS effect, Bootstrap 95% confidence interval [0.0223,0.0507], indicating that the chain mediated effect of BMI at 42 days postpartum, BMI at current was significant; third, the indirect effect composed of BMI at pre-pregnancy → BMI at current → MetS, Bootstrap 95% confidence interval [0.0845,0.1558], indicating a significant mediating effect of BMI at current. The total indirect effect accounted for 80.95% of the total effect.

## Discussion

This study demonstrates that the BMI of MetS group in all stages was higher than the non-MetS group. There were significant positive correlations between BMI in each stage and ORs of MetS during lactation (*P* < 0.05). The best cut-off values for BMI in the four stages were 23.47, 30.49, 26.04 and 25.47 kg/m^2^. The mediation effect of all chains have to work through lactation BMI. BMI during lactation plays a key role in the risk of MetS. This study has theoretical and practical implications for guiding women of childbearing age to maintain a healthy BMI and prevent MetS.

Elevated body mass index (BMI) throughout pregnancy has been identified as an autonomous determinant for the emergence of metabolic syndrome (MetS) in lactating women. Li's investigation [19] revealed that pre-pregnancy BMI and weight gain during pregnancy were associated with an increased risk of MetS during pregnancy. Additionally, Zhang's research [20, 21] demonstrated that advanced maternal age heightened the likelihood of adverse pregnancy outcomes, underscoring the significance of weight management during pregnancy for elderly parturient women. Notably, excessive BMI constitutes a crucial independent risk factor for metabolic disorders [22, 23], and weight reduction has the potential to mitigate this risk. Previous studies have shown that the prevalence of MetS in mid-pregnancy women was about 12% [24]. However, compared to the general adult population, studies on MetS in pregnant women are still scarce [25–27]. Previous studies have shown that continuous childbearing would lead to the development of obesity [28–30]. It was found that increased risk of MetS in people with multiple births [31–36]. In this study, we found that elevated BMI during pre-pregnancy, prenatal period, 42 days postpartum, and current lactation stage were independent risk factors for MetS during lactation, and elevated BMI during the current lactation stage had the most harmful effect on MetS during lactation. In addition, we present the BMI cut-off values at each stage for prevention of MetS during lactation.

The risk of developing Metabolic Syndrome (MetS) in lactating women can be attributed to a high body mass index (BMI) both before pregnancy and during the postpartum period. Previous research has indicated that obesity may serve as a triggering factor for MetS, which aligns with the findings of this study [37]. Our investigation revealed that the BMI of individuals with MetS was consistently higher than that of those without MetS across all stages: pre-pregnancy, pre-labor, 42 days postpartum, and during lactation. This suggests that an increased BMI at any of these stages poses a risk for developing MetS during lactation. Consequently, it is crucial to provide heightened attention and encourage lifestyle modifications for women of reproductive age who have an elevated BMI. Also, the difference value between current BMI and BMI at 42 days postpartum is an independent risk factor for developing MetS. Although BMI as an indicator of obesity was closely related to the development of MetS, a one-time measurement did not represent the change in BMI over time. In our study, tracking different periods of BMI more comprehensively reflected the relationship between BMI and MetS in lactating women.

The role of BMI during lactation in the risk of MetS is of paramount importance. This study highlights the substantial influence of pre-pregnancy BMI on MetS, which is primarily mediated by BMI during lactation. While being overweight before and during pregnancy is associated with the development of MetS, maintaining a controlled BMI during lactation effectively prevents its occurrence, thus emphasizing the significant impact of lactation BMI on MetS. Although the precise mechanisms underlying the relationship between BMI changes and MetS remain incompletely elucidated, it is widely believed that insulin resistance plays a central role in the development of MetS [38–40]. The levels of ferritin in women undergo significant fluctuations during pre-pregnancy, prenatal, 42 days postpartum, and current lactation. Excessive iron accumulation has been found to be linked to diminished insulin secretion and the onset of type 2 diabetes [41], although the impact of metabolic disorders on serum ferritin levels remains a subject of debate [42]. BMI trajectories not only signify alterations in body dimensions, but more significantly indicate modifications in diverse nutritional metabolic pathways within the body, which are intricately associated with the development of MetS.

The association between BMI and metabolic syndrome (MetS) in lactating women may involve diverse mechanisms. Firstly, the elevation of BMI in lactating women may induce endocrine disruptions, perturbing hormonal balance and precipitating further metabolic aberrations, thereby augmenting the risk of MetS [43, 44]. Secondly, hormonal fluctuations during lactation could play a pivotal role, given that alterations in hormone levels during lactation may contribute to changes in metabolic rate and fat distribution [45]. Simultaneously, lactating women may experience alterations in diet and lifestyle, resulting in an imbalance between energy intake and expenditure, influencing the association between BMI and MetS [46]. Furthermore, lactation may modulate MetS-related physiological processes by impacting insulin sensitivity, adipocyte function, and inflammatory levels, thereby further influencing the interplay with BMI [14, 47].

Our study has significant public health implications. Based on the incidence of MetS in lactating women, we explore the relationship between pre-pregnancy, prenatal and 42 days postpartum, current lactation BMI and the risk of MetS in lactating women and provide data to clarify the risk factors associated with MetS in lactating women and to develop prevention strategies.

Our research has made significant breakthroughs in several key areas: Firstly, in contrast to previous studies that typically focused on a single time point, our research has investigated the relationship between Metabolic Syndrome (MetS) risk and BMI across four pivotal stages: pre-pregnancy, pregnancy, 42 days postpartum, and lactation. This comprehensive time-series analysis provides a more intricate understanding of the dynamic interplay between BMI and MetS risk during the lactation period. Secondly, we have underscored the critical role of BMI during lactation in modulating MetS risk. This is a novel insight that aids in a deeper comprehension of the underlying mechanisms connecting lactation, BMI, and MetS. Finally, we have included data from 1,870 lactating women from the "China Child and Lactating Mother Nutrition Health Surveillance (2016–2017)" database, providing us with a robust and diverse sample, thereby enhancing the applicability of our research findings to a broader population. These novel aspects collectively contribute to the advancement of knowledge in the field of lactation-related MetS risk assessment and offer valuable insights for both clinical practice and future research endeavors.

There are also some limitations in this study. On the one hand, the study used a questionnaire design, and the conclusions of the study are subject to selection bias. Second, the study population had less lifestyle information, and more accurate sample information will be needed in the future to fully validate the association between BMI and MetS and it is necessary to determine its molecular mechanisms.Third, it's important to note that we lacked specific data on participants' daily energy intake, which limits our ability to consider it as a covariate in our analysis. Additionally, Since pre-pregnancy health status was mainly obtained through questionnaires, the data we collected were not enough to support the clinical determination of MetS before pregnancy, although pre-pregnancy hyperglycemia and hypertension were also excluded. However, according to the observation of a large sample, the prevalence rate of MetS before pregnancy was extremely low. Therefore, after discussion by the expert group, The absence of information on whether preconception was MetS was a limitation in our study, but had limited impact on overall results. Finally, this study's findings are limited to a specific population of Chinese women, and future research should explore broader populations for generalizability. Longitudinal investigations spanning various lactation and postpartum stages would provide a more comprehensive understanding of the dynamic relationship between BMI and MetS risk.

## Conclusion

Our findings suggest that the BMI of the MetS group in all stages was higher than the non-MetS group. High BMI in all stages of pregnancy and postpartum were risk factors for MetS in lactation. The best cut-off values for BMI in the four stages were 23.47, 30.49, 26.04 and 25.47 kg/m^2^. The risk of MetS in lactating women is due to a high BMI before pregnancy and postpartum. BMI during lactation plays a key role in the risk of MetS.

## Data Availability

The datasets generated and/or analyzed during the current study are not publicly available due to confidentiality reasons. Data can be made available upon reasonable request from Lianlong Yu (lianlong00a@163.com).
